# Gratitude and Mortality Among Older US Female Nurses

**DOI:** 10.1001/jamapsychiatry.2024.1687

**Published:** 2024-07-03

**Authors:** Ying Chen, Olivia I. Okereke, Eric S. Kim, Henning Tiemeier, Laura D. Kubzansky, Tyler J. VanderWeele

**Affiliations:** 1 Human Flourishing Program, Harvard Institute for Quantitative Social Science, Cambridge, Massachusetts; 2 Department of Epidemiology, Harvard T.H. Chan School of Public Health, Boston, Massachusetts; 3 Department of Psychiatry, Massachusetts General Hospital and Harvard Medical School, Boston; 4 Channing Division of Network Medicine, Department of Medicine, Brigham and Women’s Hospital and Harvard Medical School, Boston, Massachusetts; 5 Department of Psychology, University of British Columbia, Vancouver, British Columbia, Canada; 6 Department of Social and Behavioral Sciences, Harvard T.H. Chan School of Public Health, Boston, Massachusetts

## Abstract

**Importance:**

Supporting healthy aging is a US public health priority, and gratitude is a potentially modifiable psychological factor that may enhance health and well-being in older adults. However, the association between gratitude and mortality has not been studied.

**Objective:**

To examine the association of gratitude with all-cause and cause-specific mortality in later life.

**Design, Setting, and Participants:**

This population-based prospective cohort study used data from self-reported questionnaires and medical records of 49 275 US older female registered nurses who participated in the Nurses’ Health Study (2016 questionnaire wave to December 2019). Cox proportional hazards regression models estimated the hazard ratio (HR) of deaths by self-reported levels of gratitude at baseline. These models adjusted for baseline sociodemographic characteristics, social participation, physical health, lifestyle factors, cognitive function, and mental health. Data analysis was conducted from December 2022 to April 2024.

**Exposure:**

Gratitude was assessed with the 6-item Gratitude Questionnaire, a validated and widely used measure of one’s tendency to experience grateful affect.

**Main Outcomes and Measures:**

Deaths were identified from the National Death Index, state statistics records, reports by next of kin, and the postal system. Causes of death were ascertained by physicians through reviewing death certificates and medical records.

**Results:**

Among the 49 275 participants (all female; mean [SD] age at baseline, 79 [6.16] years), 4608 incident deaths were identified over 151 496 person-years of follow-up. Greater gratitude at baseline was associated with a lower hazard of mortality in a monotonic fashion. For instance, the highest tertile of gratitude, compared with the lowest tertile, was associated with a lower hazard of all-cause deaths (HR, 0.91; 95% CI, 0.84-0.99) after adjusting for baseline sociodemographic characteristics, social participation, religious involvement, physical health, lifestyle factors, cognitive function, and mental health. When considering cause-specific deaths, death from cardiovascular disease was inversely associated with gratitude (HR, 0.85; 95% CI, 0.73-0.995).

**Conclusions and Relevance:**

This study provides the first empirical evidence suggesting that experiencing grateful affect is associated with increased longevity among older adults. The findings will need to be replicated in future studies with more representative samples.

## Key Points

**Question:**

Do people who more frequently notice and feel grateful for positive experiences tend to live longer?

**Findings:**

In this cohort study of older US female nurses, experiencing more grateful affect was associated with lower mortality. Individuals in the highest tertile of gratitude, compared with the lowest tertile, had a 9% lower hazard of deaths from any cause, after accounting for baseline sociodemographic characteristics, social participation, religious involvement, physical health, lifestyle factors, cognitive function, and mental health.

**Meaning:**

The findings suggest that the experience of grateful affect is associated with increased longevity among older adults.

## Teaser

This cohort study examines the association of gratitude with all-cause and cause-specific mortality in later life among US female registered nurses participating in the Nurses’ Health Study.

## Introduction

Promoting healthy aging is a US public health priority.^1^ While most biomedical and public health efforts to increase longevity have focused on reducing risk factors for illness, there is increasing interest in positive psychological factors that enhance health and well-being.^2^ Insights spanning from ancient wisdom to modern social science research suggest that gratitude is a potentially modifiable psychological factor that contributes to better health states.^3,4^

Gratitude can be conceptualized as both a trait and a state. Trait or dispositional gratitude is often defined as “a generalized tendency to notice and respond with grateful emotion to the positive experiences in life.”^3^ Individuals with a greater grateful disposition are more likely to experience grateful emotional states at a given time. Gratitude involves a 2-step process: first, individuals recognize obtaining a positive experience or outcome; second, individuals attribute this positive outcome to an external source (ie, gratitude can be directed toward other people, the circumstances, or intangible entities).^4^ The broaden and build theory posits that positive emotions, such as gratitude, encourage thoughts and actions (eg, sense of purpose, healthy lifestyles, prosocial behaviors). These activities foster the growth of psychological, physical, and social resources, which initiates a positive spiral that leads to improved health.^5^ While one’s tendency to experience gratitude is relatively stable, evidence from randomized clinical trials suggests that it is potentially modifiable. Simple and low-cost techniques for enhancing gratitude have been developed.^6^ However, their effectiveness in improving health, especially physical health outcomes, remains unclear.^3,6^

Prior observational studies suggest that gratitude is positively associated with health and well-being.^3^ Recent reviews and meta-analyses found that higher gratitude is associated with greater emotional well-being,^7^ a lower risk of depression,^8^ greater social well-being,^6,9^ healthier profiles of biomarkers,^10,11^ and better sleep quality.^6^ In comparison, experimental studies with interventions aimed at increasing gratitude often found small to moderate effects on improving emotional and social well-being, mixed evidence of reduced psychopathology, and some evidence for healthier cardiovascular biomarkers and better sleep quality.^3,6^ Prior work has some limitations. Many observational studies are cross-sectional with small samples and limited control for confounding.^6^ Despite theories suggesting that gratitude contributes to healthier lives, empirical evidence documenting the gratitude–physical health connection, especially with objective measures of physical health, is scant.^5^ Further, to our knowledge, the association between gratitude and mortality has not been studied.

To begin addressing these knowledge gaps, this study examined the association between gratitude and mortality in a large cohort of older women, with extensive control for potential confounding. We hypothesized that the experience of grateful affect is inversely associated with all-cause and cause-specific mortality.

## Methods

### Study Population

This study used data from the Nurses’ Health Study (NHS). NHS is an ongoing cohort established in 1976 that enrolled 121 701 US married female nurses aged 30 to 55 years.^12^ Since then, the participants have been followed up biennially with questionnaires that collected data on health, lifestyle, and psychosocial factors. The response rate exceeds 90% in each follow-up cycle. Gratitude was first assessed in the 2016 questionnaire (participants’ mean [SD] age, 79 [6.27] years); we considered this year as the baseline for the present study. Follow-up for mortality continued through December 2019. Participants who did not respond to the 2016 questionnaire, those who died before the baseline, and those with missing data on gratitude were excluded, which yielded a sample of 49 275 individuals. The participants were followed up from return of the 2016 baseline questionnaire until death, loss to follow-up, or the end of follow-up, whichever came first. Data analysis was conducted from December 2022 to April 2024. The study protocol was approved by the institutional review boards of the Brigham and Women’s Hospital and Harvard T.H. Chan School of Public Health, and those of participating registries as required. Return of the questionnaire indicated written informed consent. This study followed the Strengthening the Reporting of Observational Studies in Epidemiology (STROBE) reporting guideline.

### Assessment of Gratitude

Gratitude was measured with the previously validated 6-item Gratitude Questionnaire (GQ-6).^13^ GQ-6 measures one’s disposition to experience grateful affect (an example item: “I have so much in life to be thankful for”; see the full scale in eTable 1 in Supplement 1). Response options ranged from 1 (strongly disagree) to 7 (strongly agree). Negatively worded items were reverse coded, so that a higher score indicated greater gratitude. Participants with missing data on only 1 item were imputed with the mean value of the other 5 items; those with missing data on more than 1 item were considered as missing on gratitude and were excluded from all analyses. An overall score was derived by summing responses across items. To assess potential threshold effects, tertiles of the gratitude score were used as the primary exposure variable. As a sensitivity analysis, we reanalyzed the models using the continuous gratitude score. The GQ-6 scale has shown evidence of reliability and validity in prior studies.^7,14^ Its internal consistency is high in this sample (Cronbach α = .81).

### Ascertainment of Mortality and Cause of Mortality

Deaths were identified from the National Death Index, state statistics records, reports by next of kin, and the postal system. We were able to ascertain more than 98% of the deaths through these methods.^15^ Cause of death was identified by physicians via reviewing death certificates and medical records. We assessed all-cause mortality and cause-specific deaths due to cardiovascular disease (*International Classification of Diseases, Eighth Revision*^16^ codes 390-458), cancer (codes 140-207), respiratory diseases (codes 460-519), neurodegenerative disease (codes 290, 340, 342, and 348), infection (codes 000-136), injury (codes E800-E999), and other causes.

### Assessment of Covariates

Covariates were assessed (mostly through self-report) in the 2016 baseline questionnaire wherever data were available or at the most recent questionnaire wave prior to 2016. The sociodemographic covariates included age, race and ethnicity (race and ethnicity were measured to assess potential disparities, with acknowledgment that race is a social construct; because >95% of the participants self-identified as non-Hispanic White, race and ethnicity were categorized as non-Hispanic White or other [American Indian or Alaska Native, Asian, Black or African American, Hispanic, Native Hawaiian or Other Pacific Islander, and multiracial]), marital status, geographic region, educational level, census-tract household median income and college education rate (both based on geocoded data), retirement status, living arrangement, and special residential setting (nursing home, senior or retirement housing or community, or assisted living facility). Because community participation and spiritual involvement have been linked with gratitude and mortality in prior studies,^17,18^ we also adjusted for baseline social participation, religious service attendance, and religious coping. In addition, we adjusted for baseline physical health and lifestyle factors, including history of heart disease, stroke, cancer, high cholesterol, hypertension, and diabetes; cigarette smoking; alcohol intake; physical activity (measured in metabolic equivalents)^19^; body mass index; and dietary quality (assessed with the Alternative Healthy Eating Index^20^). Furthermore, we adjusted for baseline cognitive function (assessed with a previously validated 7-item measure of subjective cognitive decline^21^), mental health (clinician’s diagnosis of depression, antidepressant use, and depressive symptoms [assessed with the 15-item Geriatric Depression Scale^22^]), and psychological well-being (optimism, measured with the Revised Life Orientation Test^23^).

### Statistical Analysis

Cox proportional hazards models (age in months as the timescale, stratified by calendar time) were used to estimate the hazard ratios (HRs) for all-cause mortality by tertiles of gratitude at baseline. The base model adjusted for sociodemographic factors, social participation, and religious involvement; the second model additionally included baseline physical health; the third model additionally adjusted for baseline lifestyle factors; and the fully adjusted model additionally adjusted for baseline cognitive function, depression diagnosis, antidepressant use, depressive symptoms, and optimism. As a secondary analysis, we reanalyzed the fully adjusted model with cause-specific deaths as the outcome variables. Multiple imputation^24,25^ (with 10 imputed datasets created) was performed to impute missing data on covariates (missing data ranged from 0%-10.4%).

Several sensitivity analyses were performed. First, to assess potential departures from the proportional hazards model assumption, we examined the interaction terms between gratitude and age. Second, to reduce concerns about potential reverse causation by underlying health issues, we reanalyzed the primary models excluding participants who died during the first year of follow-up (n = 1182); we also reanalyzed the models excluding participants with a history of major chronic conditions at baseline (including cardiovascular diseases and cancer, n = 19 724); we further reanalyzed the models excluding participants with a clinician diagnosis of depression or antidepressant use at baseline (n = 8025); in addition, we stratified the primary analyses by whether the participants had any of the following conditions (n = 24 543): died within the first year of follow-up, had a history of major chronic conditions at baseline, or had a clinician diagnosis of depression or antidepressant use at baseline. Third, we reanalyzed the models using the continuous gratitude score as the exposure. Fourth, we reanalyzed the models using missing indicators for missing data on covariates and also with complete-case analyses. Fifth, we calculated E-values^26,27^ to evaluate robustness of the results to potential unmeasured confounding.

Statistical analyses were performed using SAS version 9.4 software (SAS Institute Inc). All statistical tests were 2-sided, and *P* < .05 was considered statistically significant.

## Results

The 49 275 participants had a mean (SD) age of 79 (6.16) years at baseline and were predominantly non-Hispanic White (47 630 participants [97%]). More than half the participants were married or had a partner (25 930 [53%]), attended religious services at least once per week (28 882 [59%]), and did not live alone (32 133 [66%]). The mean (range) gratitude score was 37.77 (6-42), similar to findings in other samples of older US women.^28^ Those who reported greater gratitude were slightly younger; were more likely to be married or in partnership; had higher socioeconomic status; had greater social participation, religious involvement, and optimism; and were healthier and less depressed at baseline (Table 1).

**Table 1.** Baseline Age-Adjusted Participant Characteristics by Levels of Gratitude From the 2016 Questionnaire of the Nurses’ Health Study^a^

Characteristic	Gratitude score tertile (N = 49 275)^b^		
	Lowest (n = 15 814)	Middle (n = 15 115)	Highest (n = 18 346)
Sociodemographic			
Age, mean (SD), y^c^	80.45 (6.34)	79.16 (6.08)	78.03 (5.83)
Race and ethnicity^d^			
Non-Hispanic White	96.98	96.83	96.26
Other	3.02	3.17	3.74
Marital status			
Married or in partnership	50.19	53.70	54.65
Divorced or separated	9.32	8.05	7.21
Widowed	40.49	38.24	38.14
Geographic region			
Northeast	49.73	46.65	45.00
Midwest	16.20	17.42	18.44
South	20.60	21.81	22.00
West	13.47	14.12	14.56
Educational level			
RN	93.48	92.83	91.80
Bachelor’s degree	3.84	4.00	4.54
Graduate degree	2.67	3.16	3.66
Census-tract household median income, $			
<50 000	25.90	25.13	25.24
50 000-74 999	49.42	48.81	48.05
75 000-99 999	18.42	18.86	19.47
≥100 000	6.26	7.19	7.23
Census-tract college education rate, mean (SD)^e^	0.31 (0.15)	0.31 (0.15)	0.32 (0.16)
Retired	92.14	90.81	89.15
Live alone	34.91	34.40	34.02
Special residential setting	10.06	9.40	8.29
Social participation and religious involvement			
Social participation, h/wk			
0	35.13	23.59	19.53
1-2	22.95	22.64	20.04
3-5	24.33	27.74	27.71
6-10	11.92	16.91	19.31
≥11	5.67	9.12	13.41
Religious service attendance			
Never or almost never	36.20	26.34	21.51
Less than once/wk	14.43	13.15	12.50
At least once/wk	49.37	60.51	65.99
Religious coping			
Not at all	16.68	10.50	8.87
Not very	13.43	9.11	5.88
Somewhat	34.45	28.86	21.40
Very	35.45	51.53	63.85
Physical health history			
Heart disease	14.74	12.41	11.63
Stroke	6.03	4.49	4.00
Cancer	29.42	28.24	28.63
High cholesterol	81.89	79.32	77.63
Hypertension	76.76	73.03	70.22
Diabetes	18.99	16.00	14.39
Lifestyle			
Cigarette smoking			
Never	43.56	46.55	49.60
Former	52.48	50.65	47.86
Current, No. of cigarettes/d			
1-14	2.56	1.78	1.76
15-24	1.20	0.80	0.64
≥25	0.21	0.22	0.13
Alcohol intake, g/d			
0	45.20	41.98	42.02
0.1-9.9	32.88	34.88	34.29
10.0-29.9	16.56	18.02	18.30
≥30.0	5.36	5.12	5.38
Physical activity, METS			
<3.0	21.19	14.42	12.44
3.0-8.9	23.22	21.05	18.89
9.0-17.9	19.38	19.93	19.24
18.0-26.9	11.93	14.38	14.10
≥27.0	24.28	30.22	35.33
BMI			
<20.0	10.31	9.46	9.43
20.0-24.9	35.87	38.31	39.33
25.0-29.9	31.91	32.30	32.94
30.0-34.9	14.45	13.88	12.74
≥35.0	7.47	6.04	5.55
Dietary quality score, mean (SD)^f^	60.47 (12.00)	62.27 (11.88)	63.48 (12.20)
Cognitive function, mental health, and well-being^g^			
Subjective cognitive decline, mean (SD)	1.06 (1.40)	0.84 (1.23)	0.67 (1.11)
Clinician diagnosis of depression or antidepressant use	21.21	15.89	12.66
Depressive symptoms, mean (SD)	2.88 (2.88)	1.55 (1.78)	1.04 (1.45)
Optimism, mean (SD)	16.58 (4.57)	18.87 (4.03)	20.44 (3.71)

Abbreviations: BMI, body mass index (calculated as weight in kilograms divided by height in meters squared); METS, metabolic equivalents; RN, registered nurse.

^a^ Values for continuous variables are shown as mean (SD); all other values are for categorical variables and are presented as percentages. Values are standardized to the age distribution of the study population. Values of polytomous variables may not sum to 100% due to rounding.

^b^ The 6-item Gratitude Questionnaire (possible score range, 6-42) was used to assess one’s disposition to experience grateful affect. The score range for the lowest tertile was 6 to 36; middle tertile, 37 to 40; and highest tertile, 41 to 42.

^c^ Value is not age adjusted. Ages ranged from 69 to 96 years.

^d^ Race and ethnicity were self-reported. Because more than 95% of the participants self-identified as non-Hispanic White, race and ethnicity were categorized as non-Hispanic White or other. Other race and ethnicity included American Indian or Alaska Native, Asian, Black or African American, Hispanic, Native Hawaiian or Other Pacific Islander, and multiracial.

^e^ Range, 0.02 to 0.88.

^f^ Range, 22 to 101.

^g^ Ranges were 0 to 7 for subjective cognitive decline, 0 to 15 for depressive symptoms, and 0 to 24 for optimism.

There were 4608 incident deaths from any causes identified over 151 496 person-years of follow-up (mean [SD] follow-up, 3.07 [0.57] years). These included 1364 deaths from cardiovascular disease, 273 from cancer, 406 from respiratory disease, 492 from neurodegenerative disease, 114 from infection, 70 from injury, and 1889 from other causes (Table 2 and Table 3).

**Table 2.** Associations Between Gratitude and All-Cause Mortality Among 49 275 Participants in the Nurses’ Health Study, 2016-2019

Model	Gratitude score tertile, HR (95% CI)^a^			*P* value for trend
	Lowest	Middle	Highest	
1. Adjusted for demographic, social, and religious factors^b^	1 [Reference]	0.77 (0.72-0.83)	0.71 (0.66-0.76)	<.001
2. Further adjusted for physical health^c^	1 [Reference]	0.79 (0.74-0.85)	0.73 (0.68-0.79)	<.001
3. Further adjusted for health behaviors^d^	1 [Reference]	0.83 (0.77-0.89)	0.79 (0.73-0.85)	<.001
4. Further adjusted for cognitive function, mental health, and well-being^e^	1 [Reference]	0.93 (0.86-1.01)	0.91 (0.84-0.99)	.02

Abbreviation: HR, hazard ratio.

^a^ There were 2153 deaths and 47 413 person-years among participants in the lowest gratitude score tertile, 1273 deaths and 46 790 person-years among those in the middle tertile, and 1182 deaths and 57 293 person-years among those in the highest tertile. For HR models, multiple imputation was performed to impute missing data on covariates.

^b^ Model 1 controlled for age, race and ethnicity, marital status, geographic region, educational level, census-tract median household income, census-tract college education rate, retired, living arrangement, special residential setting, social participation, religious service attendance, and religious coping.

^c^ Model 2 included all covariates in model 1 plus baseline physical health status, including history of heart disease, stroke, cancer, hypertension, hypercholesterolemia, and diabetes.

^d^ Model 3 included all covariates in model 2 plus baseline health behaviors, including alcohol intake, smoking status, body mass index, physical activity, and the dietary quality score (measured with the 2010 Alternative Healthy Eating Index).

^e^ Model 4 included all covariates in model 3 plus baseline cognitive function, mental health, and psychological well-being, including subjective cognitive decline, clinician diagnosis of depression or antidepressant use, depressive symptoms, and optimism.

**Table 3.** Associations Between Gratitude and Cause-Specific Mortality Among 49 275 Participants in the Nurses’ Health Study, 2016-2019

Cause of death	Deaths, No.	Gratitude score tertile, HR (95% CI)^a^			*P* value for trend
		Lowest	Middle	Highest	
Cardiovascular disease	1364	1 [Reference]	0.93 (0.81-1.06)	0.85 (0.73-0.995)	.04
Cancer	273	1 [Reference]	0.96 (0.72-1.27)	0.87 (0.69-1.11)	.26
Respiratory disease	406	1 [Reference]	0.93 (0.80-1.09)	0.86 (0.72-1.02)	.08
Neurodegenerative disease	492	1 [Reference]	0.91 (0.72-1.14)	0.83 (0.66-1.06)	.16
Infection	114	1 [Reference]	0.91 (0.72-1.16)	0.90 (0.60-1.36)	.58
Injury	70	1 [Reference]	0.90 (0.64-1.25)	0.84 (0.62-1.13)	.27
Other	1889	1 [Reference]	0.93 (0.81-1.07)	0.87 (0.73-1.03)	.10

Abbreviation: HR, hazard ratio.

^a^ Multiple imputation was performed to impute missing data on covariates. All analyses adjusted for all the following covariates: age, race and ethnicity, marital status, geographic region, educational level, census-tract median household income, census-tract college education rate, retired, living arrangement, special residential setting, social participation, religious service attendance, religious coping, history of heart disease, stroke, cancer, hypertension, hypercholesterolemia, diabetes, alcohol intake, smoking status, body mass index, physical activity, dietary quality score (measured with the 2010 Alternative Healthy Eating Index), subjective cognitive decline, clinician diagnosis of depression or antidepressant use, depressive symptoms, and optimism.

The multivariable-adjusted proportional hazards analyses suggested an inverse monotonic association between gratitude and all-cause mortality (Table 2). The base model showed that the highest tertile of gratitude, compared with the lowest tertile, was associated with a lower hazard of all-cause deaths (HR, 0.71; 95% CI, 0.66-0.76), adjusting for sociodemographic factors, social participation, and religious involvement. The association remained after additionally adjusting for baseline physical health, lifestyle factors, cognitive function, and mental health and well-being (eg, in the fully adjusted model, HR, 0.91; 95% CI, 0.84-0.99). We found no evidence suggesting violations of the proportional hazards assumption. The point estimates were similar (although 95% CIs became wider, which was likely due to reduced sample sizes) when the analyses excluded participants who died during the first year of follow-up (eTable 2 in Supplement 1), excluded participants with a history of major chronic diseases at baseline (eTable 3 in Supplement 1), and excluded those with a clinician diagnosis of depression or antidepressant use at baseline (eTable 4 in Supplement 1). In the sensitivity analysis stratified by whether the participants had died during the first year of follow-up, had major chronic diseases at baseline, or had a clinician diagnosis of depression or antidepressant use at baseline, point estimates were similar between those with and without these conditions (eTable 5 in Supplement 1). Sensitivity analysis with the continuous gratitude score also yielded similar results (eTable 6 in Supplement 1). Analyses using missing indicators for missing data on covariates and analyses of complete cases both yielded point estimates similar to those of the primary analyses (eTables 7 and 8 in Supplement 1).

The secondary analysis, which focused on cause-specific mortality, showed that death from cardiovascular disease was inversely associated with gratitude (HR, 0.85; 95% CI, 0.73-0.995) (Table 3). The associations between gratitude and remaining causes of death did not reach *P* < .05. The 95% CIs were wide due to a small number of deaths for each cause.

The calculation of E-values^26,27^ suggested that the observed associations between gratitude and mortality were moderately robust to potential unmeasured confounding. To explain away an HR of 0.91 for all-cause deaths, an unmeasured confounder associated with both increased likelihood of gratitude and decreased likelihood of deaths by risk ratios of 1.43 each, above and beyond all measured covariates, could suffice but weaker joint confounder associations could not. Similarly, to shift the 95% CIs to include the null value, an unmeasured confounder associated with increased gratitude and decreased deaths by risk ratios of 1.11-fold each could suffice, but weaker joint confounder association could not. To provide further context for understanding the magnitude of the E-values, we reported the associations between each measured covariate and mortality from the fully adjusted model in eTable 9 in Supplement 1.

## Discussion

Gratitude is often considered a positive emotion that may be desirable in its own right.^5^ This study provides the first empirical evidence to suggest that gratitude may be a psychological resource associated with increased longevity in later life.

The literature suggests that gratitude is inversely associated with risk factors of mortality. For instance, greater gratitude was associated with favorable profiles of cardiovascular biomarkers, such as endothelial function, prognostic inflammatory markers, and lipids,^10,11^ and greater adherence to healthy lifestyles (eg, diet, exercise, medication adherence).^10,29^ A recent meta-analysis of longitudinal studies also found an inverse association between gratitude and depression (*r* = −0.33).^8^ In addition, gratitude was correlated with other facets of psychosocial functioning, such as greater purpose in life and social support, which have been linked with increased longevity.^7,30,31^ Prior researchers have developed theoretical models to understand potential mechanisms underlying gratitude and health.^32^ It was posited that gratitude can shape health directly via increasing restorative biological functioning and healthy lifestyles as well as indirectly through increased social support, prosociality, and adaptive coping strategies.^32^ Of note, many of these factors may have bidirectional associations with gratitude^33,34^ and could potentially confound the gratitude and mortality association. Because this study measured these factors only before or at the same time when gratitude was assessed, but not afterward, we controlled for these factors as potential confounders and cannot perform causal mediation analyses.

Experiencing gratitude may be particularly relevant for generating a sense of meaning and connectedness in older adults, which facilitates coping with aging-related changes.^35^ The socioemotional selective theory^36^ suggests that people become increasingly aware that their lifetime is limited as they age. This awareness leads older adults to prioritize engagement in meaningful events and close relationships. To this end, experiencing and expressing gratitude is a contributor that helps maintain meaningful social bonds.^37^ While self-reported levels of gratitude generally increase with age, some studies suggest that gratitude reaches a plateau and starts declining among older-old and oldest-old individuals.^35,38^ However, evidence on the association between gratitude and health in older-old and oldest-old individuals remains limited. This study adds evidence on gratitude and longevity in these age groups.

While gratitude is a universal human experience, it can have a profound spiritual grounding. In many religious communities, positive aspects of life, such as health and love, are seen as gifts with appreciation. This perspective motivates individuals to practice self-care and prosocial activities and to grow via meaning-making in the midst of adversity.^39,40^ Some facets of religious involvement may be associated with reduced risks of mortality and morbidity.^41^ However, prior observational studies on gratitude and health have not often accounted for potential confounding by religion. This study adjusted for baseline religious service attendance and religious coping in all analyses.

The associations reported in this study constitute averages and suggest average beneficial longevity outcomes associated with gratitude. However, this may not be the case for every individual. Some researchers hypothesized that gratitude may have adverse effects on well-being under certain situations.^42^ For instance, gratitude often involves a feeling that kindness is an altruistic gift from others that can never be fully repaid. This sometimes leads to a sense of indebtedness,^6,39^ which may affect one’s autonomy and reinforce hierarchical relationships.^40^ We do not have data to examine these hypotheses. However, these potential negative impacts of gratitude warrant further investigation in future studies.

### Limitations

This study has some limitations. First, the GQ-6 scale is a widely used measure that focuses on assessing grateful affect. However, gratitude is sometimes considered a multifaceted construct that comprises other aspects, such as the behavioral expression of gratitude.^3^ Future studies that use multifactorial measures of gratitude (eg, Gratitude Resentment and Appreciation Test,^43^ Gratitude Questionnaire–20 Items^44^) could further elucidate which aspects of gratitude are more closely associated with mortality. Second, to reduce potential confounding, we took a conservative approach adjusting for a wide range of covariates. Some covariates (eg, religious coping, optimism) may have overlapping components with gratitude^45,46^ or may be potential mediators for the gratitude-mortality association (eg, depressive symptoms, lifestyle factors), and control for these may constitute overadjustment. It is, however, striking that the association between gratitude and reduced mortality persists even after adjustment for this stringent set of covariates. Next, the participants are all US female nurses and are primarily non-Hispanic White and of Christian denomination, which limits generalizability of the results to other populations. The participants were also considerably older, and associations at younger ages might be different. Some prior evidence suggests that the gratitude and well-being association may vary by age, culture, and country-level collectivism orientation.^7^ It would be worthwhile to replicate this study in other sociodemographic, religious, and cultural groups. These limitations are balanced by important contributions of this study. It is the first, to our knowledge, to evaluate the gratitude and mortality association; further, the study was conducted in a large sample, with rigorous control for potential confounding.

## Conclusions

Low-cost and easy-to-implement techniques that aim to enhance gratitude are available.^3,6^ Examples include writing on a regular basis about people or things that one feels grateful for (eg, gratitude journaling, the Three Good Things exercise), grateful contemplation, and behavioral expression of gratitude (eg, the gratitude visit exercise). However, their effects on improving health outcomes, especially physical health outcomes, remain unclear.^6,47^ While this study provides preliminary evidence for an inverse association between gratitude and mortality among older female nurses, the findings will need to be replicated in future studies with more representative samples. Gratitude is generally considered a positive emotion in its own right, and as evidence accumulates, there will be better understanding of its role in enhancing health and longevity.

## Notes

Supplement 1. **eTable 1.** The 6-Item Gratitude Questionnaire (GQ-6) **eTable 2.** Associations Between Gratitude and All-Cause Mortality, Excluding Participants Who Died Within the First Year of Follow-Up (Nurses’ Health Study 2016-2019, N=48,093) **eTable 3.** Associations Between Gratitude and All-Cause Mortality, Excluding Participants With Major Chronic Diseases at Baseline (Nurses’ Health Study 2016-2019, N=29,551) **eTable 4.** Associations Between Gratitude and All-Cause Mortality, Excluding Participants With a Clinician Diagnosis of Depression or Antidepressant Use at Baseline (Nurses’ Health Study 2016-2019, N=41,250) **eTable 5.** Associations Between Gratitude and All-Cause Mortality, Stratified by Whether the Participants Had Any of the Following Conditions: Died Within the First Year of Follow-Up, Had Major Chronic Diseases at Baseline, or Had a Clinician Diagnosis of Depression or Antidepressant Use at Baseline (Nurses’ Health Study 2016-2019, N=49,275) **eTable 6.** Associations Between the Continuous Gratitude Score and All-Cause Mortality (Nurses’ Health Study 2016-2019, N=49,275) **eTable 7.** Associations Between Gratitude and All-Cause Mortality, Using Missing Indicators for Missing Data on the Covariates (Nurses’ Health Study 2016-2019, N=49,275) **eTable 8.** Complete-Case Analysis on the Associations Between Gratitude and All-Cause Mortality (Nurses’ Health Study 2016-2019, N=32,706) **eTable 9.** Associations Between Covariates and All-Cause Mortality in the Fully Adjusted Model (Nurses’ Health Study 2016-2019, N=49,275)

Supplement 2. Data Sharing Statement
