# Supplementary material for: Gratitude and Mortality Among Older US Female Nurses
Source: JAMA Psychiatry. 2024 Jul 3;81(10):1030–8. doi: 10.1001/jamapsychiatry.2024.1687 (PMC11223047; doi:10.1001/jamapsychiatry.2024.1687)
Supplement: Supplement 1. — eTable 1. The 6-Item Gratitude Questionnaire (GQ-6) eTable 2. Associations Between Gratitude and All-Cause Mortality, Excluding Participants Who Died Within the First Year of Follow-Up (Nurses’ Health Study 2016-2019, N=48,093) eTable 3. Associations Between Gratitude and All-Cause Mortality, Excluding Participants With Major Chronic Diseases at Baseline (Nurses’ Health Study 2016-2019, N=29,551) eTable 4. Associations Between Gratitude and All-Cause Mortality, Excluding Participants With a Clinician Diagnosis of Depression or Antidepressant Use at Baseline (Nurses’ Health Study 2016-2019, N=41,250) eTable 5. Associations Between Gratitude and All-Cause Mortality, Stratified by Whether the Participants Had Any of the Following Conditions: Died Within the First Year of Follow-Up, Had Major Chronic Diseases at Baseline, or Had a Clinician Diagnosis of Depression or Antidepressant Use at Baseline (Nurses’ Health Study 2016-2019, N=49,275) eTable 6. Associations Between the Continuous Gratitude Score and All-Cause Mortality (Nurses’ Health Study 2016-2019, N=49,275) eTable 7. Associations Between Gratitude and All-Cause Mortality, Using Missing Indicators for Missing Data on the Covariates (Nurses’ Health Study 2016-2019, N=49,275) eTable 8. Complete-Case Analysis on the Associations Between Gratitude and All-Cause Mortality (Nurses’ Health Study 2016-2019, N=32,706) eTable 9. Associations Between Covariates and All-Cause Mortality in the Fully Adjusted Model (Nurses’ Health Study 2016-2019, N=49,275) [file jamapsychiatry-e241687-s001.pdf]

Supplemental Online Content

Chen Y, Okereke OI, Kim ES, Tiemeier H, Kubzansky LD, VanderWeele TJ. Gratitude and mortality among older US female nurses. *JAMA Psychiatry*. Published online July 3, 2024. doi:10.1001.jamapsychiatry.2024.1687

eTable 1. The 6-Item Gratitude Questionnaire (GQ-6)

eTable 2. Associations Between Gratitude and All-Cause Mortality, Excluding Participants Who Died Within the First Year of Follow-Up (Nurses' Health Study 2016-2019, N=48,093)

eTable 3. Associations Between Gratitude and All-Cause Mortality, Excluding Participants With Major Chronic Diseases at Baseline (Nurses' Health Study 2016-2019, N=29,551)

eTable 4. Associations Between Gratitude and All-Cause Mortality, Excluding Participants With a Clinician Diagnosis of Depression or Antidepressant Use at Baseline (Nurses' Health Study 2016-2019, N=41,250)

eTable 5. Associations Between Gratitude and All-Cause Mortality, Stratified by Whether the Participants Had Any of the Following Conditions: Died Within the First Year of Follow-Up, Had Major Chronic Diseases at Baseline, or Had a Clinician Diagnosis of Depression or Antidepressant Use at Baseline (Nurses' Health Study 2016-2019, N=49,275)

eTable 6. Associations Between the Continuous Gratitude Score and All-Cause Mortality (Nurses' Health Study 2016-2019, N=49,275)

eTable 7. Associations Between Gratitude and All-Cause Mortality, Using Missing Indicators for Missing Data on the Covariates (Nurses' Health Study 2016-2019, N=49,275)

eTable 8. Complete-Case Analysis on the Associations Between Gratitude and All-Cause Mortality (Nurses' Health Study 2016-2019, N=32,706)

eTable 9. Associations Between Covariates and All-Cause Mortality in the Fully Adjusted Model (Nurses' Health Study 2016-2019, N=49,275)

This supplemental material has been provided by the authors to give readers additional information about their work.

© 2024 Chen Y et al. *JAMA Psychiatry.*

eTable 1. The 6-Item Gratitude Questionnaire (GQ-6)

1. I have so much in life to be thankful for.
2. If I had to list everything that I felt grateful for, it would be a very long list.
3. When I look at the world, I don't see much to be grateful for.
4. I am grateful to a wide variety of people.
5. As I get older I find myself more able to appreciate the people, events, and situations that have been part of my life history.
6. Long amounts of time can go by before I feel grateful to something or someone.

Response options ranged from 1 (strongly disagree) to 7 (strongly agree). Items 3 and 6 are reverse-scored, so that higher scores indicate greater gratitude. In this analytic sample, 98% of participation had valid data on all 6 items. Participants with missing data on only 1 of the 6 items were imputed with the mean value of the other 5 items. Participants with missing data on more than one item were considered as missing on gratitude, and they were excluded from all analyses.

© 2024 Chen Y et al. *JAMA Psychiatry.*

eTable 2. Associations Between Gratitude and All-Cause Mortality, Excluding Participants Who Died Within the First Year of Follow-Up (Nurses' Health Study 2016-2019, N=48,093)

	**Gratitude Score Tertiles**		
	Bottom tertile (reference)	Middle tertile HR (95% CI)	Top tertile HR (95% CI)
Model 1 [a]	1.00	0.78 (0.72, 0.85)***	0.72 (0.66, 0.79)***
Model 2 [b]	1.00	0.80 (0.74, 0.87)***	0.75 (0.69, 0.82)***
Model 3 [c]	1.00	0.84 (0.77, 0.91)***	0.80 (0.74, 0.88)***
Model 4 [d]	1.00	0.92 (0.84, 1.00)	0.90 (0.82, 0.99)*

Notes: HR, hazard ratio; CI, confidence interval. Multiple imputation was performed to impute missing data on the covariates. **p*<0.05, ***p*<0.01, ****p*<0.001.

a. Model 1 controlled for age (years), race/ethnicity (non-Hispanic white, others), marital status (married/in partnership, divorced/separated, widowed), geographic region (Northeast, Midwest, South, West), educational level (RN, bachelor, graduate), census-tract median household income (<$50,000, $50,000-$74,999, $75,000-$99,999, >=$100,000), census-tract college education rate (continuous), retired (yes, no), living arrangement (live alone, others), special residential setting (yes, no), social participation (none, 1-2 hours/week, 3-5 hours/week, 6-10 hours/week, 11+ hours/week), religious service attendance (never/almost never, <once/week, >=once/week), and religious coping (not at all, not very, somewhat, very).

b. Model 2 included all covariates in model 1, and additionally adjusted for baseline physical health status including history of heart disease (yes, no), stroke (yes, no), cancer (yes, no), hypertension (yes, no), hypercholesterolemia (yes, no), and diabetes (yes, no).

c. Model 3 included all covariates in model 2, and additionally adjusted for baseline health behaviors including alcohol intake (0, 0.1-9.9, 10.0-29.9, 30+ g/d), smoking status (never, former, current 1-14/d, current 15-24/day, >=25/day), body mass index (<20, 20-24.9, 25-29.9, 30-34.9, 35+ kg/m2), physical activity (<3, 3-8.9, 9-17.9, 18-26.9, ≥27 METS), and dietary quality score (continuous score).

d. Model 4 included all covariates in model 3, and additionally adjusted for baseline cognitive function, mental health and psychological well-being including subjective cognitive decline (continuous score), depression diagnosis or antidepressant use (yes, no), depressive symptoms (continuous score), and optimism (continuous score).

© 2024 Chen Y et al. *JAMA Psychiatry.*

eTable 3. Associations Between Gratitude and All-Cause Mortality, Excluding Participants With Major Chronic Diseases at Baseline (Nurses' Health Study 2016-2019, N=29,551)

	Gratitude Score Tertiles		
	Bottom tertile (reference)	Middle tertile HR (95% CI)	Top tertile HR (95% CI)
Model 1 [a]	1.00	0.76 (0.68, 0.84)***	0.75 (0.66, 0.84)***
Model 2 [b]	1.00	0.76 (0.68, 0.85)***	0.75 (0.67, 0.85)***
Model 3 [c]	1.00	0.79 (0.71, 0.88)***	0.81 (0.72, 0.91)***
Model 4 [d]	1.00	0.90 (0.80, 1.01)	0.92 (0.81, 1.05)

Notes: HR, hazard ratio; CI, confidence interval. Multiple imputation was performed to impute missing data on the covariates. The analyses excluded participants who had a history of heart diseases, stroke, or cancer at baseline. *p<0.05, **p<0.01, ***p<0.001.

a. Model 1 controlled for age (years), race/ethnicity (non-Hispanic white, others), marital status (married/in partnership, divorced/separated, widowed), geographic region (Northeast, Midwest, South, West), educational level (RN, bachelor, graduate), census-tract median household income (<$50,000, $50,000-$74,999, $75,000-$99,999, >=$100,000), census-tract college education rate (continuous), retired (yes, no), living arrangement (live alone, others), special residential setting (yes, no), social participation (none, 1-2 hours/week, 3-5 hours/week, 6-10 hours/week, 11+ hours/week), religious service attendance (never/almost never, <once/week, >=once/week), and religious coping (not at all, not very, somewhat, very).

b. Model 2 included all covariates in model 1, and additionally adjusted for baseline physical health status including history of hypertension (yes, no), hypercholesterolemia (yes, no), and diabetes (yes, no).

c. Model 3 included all covariates in model 2, and additionally adjusted for baseline health behaviors including alcohol intake (0, 0.1-9.9, 10.0-29.9, 30+ g/d), smoking status (never, former, current 1-14/d, current 15-24/day, >=25/day), body mass index (<20, 20-24.9, 25-29.9, 30-34.9, 35+ kg/m2), physical activity (<3, 3-8.9, 9-17.9, 18-26.9, ≥27 METS), and dietary quality score (continuous score).

d. Model 4 included all covariates in model 3, and additionally adjusted for baseline cognitive function, mental health and psychological well-being including subjective cognitive decline (continuous score), depression diagnosis or antidepressant use (yes, no), depressive symptoms (continuous score), and optimism (continuous score).

© 2024 Chen Y et al. *JAMA Psychiatry.*

eTable 4. Associations Between Gratitude and All-Cause Mortality, Excluding Participants With a Clinician Diagnosis of Depression or Antidepressant Use at Baseline (Nurses' Health Study 2016-2019, N=41,250)

	Gratitude Score Tertiles		
	Bottom tertile (reference)	Middle tertile HR (95% CI)	Top tertile HR (95% CI)
Model 1 [a]	1.00	0.78 (0.72, 0.84)***	0.73 (0.68, 0.80)***
Model 2 [b]	1.00	0.80 (0.74, 0.86)***	0.76 (0.70, 0.82)***
Model 3 [c]	1.00	0.83 (0.76, 0.90)***	0.81 (0.75, 0.88)***
Model 4 [d]	1.00	0.92 (0.85, 1.00)	0.92 (0.84, 1.01)

Notes: HR, hazard ratio; CI, confidence interval. Multiple imputation was performed to impute missing data on the covariates. The analyses excluded participants who had a clinician-diagnosis of depression or antidepressant use at baseline. *p<0.05, **p<0.01, ***p<0.001.

a. Model 1 controlled for age (years), race/ethnicity (non-Hispanic white, others), marital status (married/in partnership, divorced/separated, widowed), geographic region (Northeast, Midwest, South, West), educational level (RN, bachelor, graduate), census-tract median household income (<$50,000, $50,000-$74,999, $75,000-$99,999, >=$100,000), census-tract college education rate (continuous), retired (yes, no), living arrangement (live alone, others), special residential setting (yes, no), social participation (none, 1-2 hours/week, 3-5 hours/week, 6-10 hours/week, 11+ hours/week), religious service attendance (never/almost never, <once/week, >=once/week), and religious coping (not at all, not very, somewhat, very).

b. Model 2 included all covariates in model 1, and additionally adjusted for baseline physical health status including history of heart disease (yes, no), stroke (yes, no), cancer (yes, no), hypertension (yes, no), hypercholesterolemia (yes, no), and diabetes (yes, no).

c. Model 3 included all covariates in model 2, and additionally adjusted for baseline health behaviors including alcohol intake (0, 0.1-9.9, 10.0-29.9, 30+ g/d), smoking status (never, former, current 1-14/d, current 15-24/day, >=25/day), body mass index (<20, 20-24.9, 25-29.9, 30-34.9, 35+ kg/m2), physical activity (<3, 3-8.9, 9-17.9, 18-26.9, ≥27 METS), and dietary quality score (continuous score).

d. Model 4 included all covariates in model 3, and additionally adjusted for baseline subjective cognitive decline (continuous score), depressive symptoms (continuous score), and optimism (continuous score).

© 2024 Chen Y et al. *JAMA Psychiatry.*

eTable 5. Associations Between Gratitude and All-Cause Mortality, Stratified by Whether the Participants Had Any of the Following Conditions: Died Within the First Year of Follow-Up, Had Major Chronic Diseases at Baseline, or Had a Clinician Diagnosis of Depression or Antidepressant Use at Baseline (Nurses' Health Study 2016-2019, N=49,275)

	**Participants who did not die within the 1st year of follow-up, and did not have major chronic diseases, depression diagnosis, or antidepressant use at baseline (N=24,732)**		**Participants with any of the following conditions: died within the 1st year of follow-up, had major chronic diseases, depression diagnosis, or antidepressant use at baseline (N=24,543)**	
	Gratitude score Middle (vs. Lowest) tertile HR (95% CI)	Gratitude score Highest (vs. Lowest) tertile HR (95% CI)	Gratitude score Middle (vs. Lowest) tertile HR (95% CI)	Gratitude score Highest (vs. Lowest) tertile HR (95% CI)
Model 1 [a]	0.79 (0.68, 0.90)***	0.84 (0.73, 0.97)*	0.79 (0.69, 0.91)***	0.83 (0.70, 0.99)*
Model 2 [b]	0.79 (0.69, 0.91)**	0.86 (0.75, 0.99)*	0.79 (0.69, 0.91)***	0.84 (0.70, 1.01)
Model 3 [c]	0.81 (0.70, 0.94)**	0.91 (0.79, 1.05)	0.82 (0.71, 0.94)**	0.90 (0.75, 1.06)
Model 4 [d]	0.86 (0.75, 1.00)	0.96 (0.82, 1.12)	0.87 (0.75, 1.02)	0.95 (0.82, 1.12)

Notes: HR, hazard ratio; CI, confidence interval. Multiple imputation was performed to impute missing data on the covariates. *p<0.05, **p<0.01, ***p<0.001.

a. Model 1 controlled for age (years), race/ethnicity (non-Hispanic white, others), marital status (married/in partnership, divorced/separated, widowed), geographic region (Northeast, Midwest, South, West), educational level (RN, bachelor, graduate), census-tract median household income (<$50,000, $50,000-$74,999, $75,000-$99,999, >=$100,000), census-tract college education rate (continuous), retired (yes, no), living arrangement (live alone, others), special residential setting (yes, no), social participation (none, 1-2 hours/week, 3-5 hours/week, 6-10 hours/week, 11+ hours/week), religious service attendance (never/almost never, <once/week, >=once/week), and religious coping (not at all, not very, somewhat, very).

b. Model 2 included all covariates in model 1, and additionally adjusted for baseline physical health status including history of hypertension (yes, no), hypercholesterolemia (yes, no), and diabetes (yes, no).

c. Model 3 included all covariates in model 2, and additionally adjusted for baseline health behaviors including alcohol intake (0, 0.1-9.9, 10.0-29.9, 30+ g/d), smoking status (never, former, current 1-14/d, current 15-24/day, >=25/day), body mass index (<20, 20-24.9, 25-29.9, 30-34.9, 35+ kg/m2), physical activity (<3, 3-8.9, 9-17.9, 18-26.9, ≥27 METS), and dietary quality score (continuous score).

d. Model 4 included all covariates in model 3, and additionally adjusted for baseline subjective cognitive decline (continuous score), depressive symptoms (continuous score), and optimism (continuous score).

© 2024 Chen Y et al. *JAMA Psychiatry.*

eTable 6. Associations Between the Continuous Gratitude Score and All-Cause Mortality (Nurses' Health Study 2016-2019, N=49,275)

	Continuous Gratitude Score (Standardized)		
	Hazard ratio of mortality	95% confidence interval	p value
Model 1 [a]	0.86	0.84, 0.88	<.001
Model 2 [b]	0.87	0.85, 0.89	<.001
Model 3 [c]	0.90	0.87, 0.92	<.001
Model 4 [d]	0.97	0.94, 1.00	0.055

Notes: The gratitude score was standardized at mean=0 and standard deviation=1. Multiple imputation was performed to impute missing data on the covariates.

a. Model 1 controlled for age (years), race/ethnicity (non-Hispanic white, others), marital status (married/in partnership, divorced/separated, widowed), geographic region (Northeast, Midwest, South, West), educational level (RN, bachelor, graduate), census-tract median household income (<$50,000, $50,000-$74,999, $75,000-$99,999, >=$100,000), census-tract college education rate (continuous), retired (yes, no), living arrangement (live alone, others), special residential setting (yes, no), social participation (none, 1-2 hours/week, 3-5 hours/week, 6-10 hours/week, 11+ hours/week), religious service attendance (never/almost never, <once/week, >=once/week), and religious coping (not at all, not very, somewhat, very).

b. Model 2 included all covariates in model 1, and additionally adjusted for baseline physical health status including history of heart disease (yes, no), stroke (yes, no), cancer (yes, no), hypertension (yes, no), hypercholesterolemia (yes, no), and diabetes (yes, no).

c. Model 3 included all covariates in model 2, and additionally adjusted for baseline health behaviors including alcohol intake (0, 0.1-9.9, 10.0-29.9, 30+ g/d), smoking status (never, former, current 1-14/d, current 15-24/day, >=25/day), body mass index (<20, 20-24.9, 25-29.9, 30-34.9, 35+ kg/m2), physical activity (<3, 3-8.9, 9-17.9, 18-26.9, ≥27 METS), and dietary quality score (continuous score).

d. Model 4 included all covariates in model 3, and additionally adjusted for baseline cognitive function, mental health and psychological well-being including subjective cognitive decline (continuous score), depression diagnosis or antidepressant use (yes, no), depressive symptoms (continuous score), and optimism (continuous score).

© 2024 Chen Y et al. *JAMA Psychiatry.*

eTable 7. Associations Between Gratitude and All-Cause Mortality, Using Missing Indicators for Missing Data on the Covariates (Nurses' Health Study 2016-2019, N=49,275)

	Gratitude Score Tertiles		
	Bottom tertile (reference)	Middle tertile HR (95% CI)	Top tertile HR (95% CI)
Model 1 [a]	1.00	0.77 (0.72, 0.83)***	0.71 (0.66, 0.77)***
Model 2 [b]	1.00	0.79 (0.74, 0.85)***	0.73 (0.68, 0.79)***
Model 3 [c]	1.00	0.82 (0.76, 0.89)***	0.79 (0.73, 0.86)***
Model 4 [d]	1.00	0.91 (0.84, 0.99)*	0.90 (0.82, 0.98)*

Notes: HR, hazard ratio; CI, confidence interval. Missing indicators were used for missing data on the covariates. **p*<0.05, ***p*<0.01, ****p*<0.001.

a. Model 1 controlled for age (years), race/ethnicity (non-Hispanic white, others), marital status (married/in partnership, divorced/separated, widowed), geographic region (Northeast, Midwest, South, West), educational level (RN, bachelor, graduate), census-tract median household income (<$50,000, $50,000-$74,999, $75,000-$99,999, >=$100,000), census-tract college education rate (continuous), retired (yes, no), living arrangement (live alone, others), special residential setting (yes, no), social participation (none, 1-2 hours/week, 3-5 hours/week, 6-10 hours/week, 11+ hours/week), religious service attendance (never/almost never, <once/week, >=once/week), and religious coping (not at all, not very, somewhat, very).

b. Model 2 included all covariates in model 1, and additionally adjusted for baseline physical health status including history of heart disease (yes, no), stroke (yes, no), cancer (yes, no), hypertension (yes, no), hypercholesterolemia (yes, no), and diabetes (yes, no).

c. Model 3 included all covariates in model 2, and additionally adjusted for baseline health behaviors including alcohol intake (0, 0.1-9.9, 10.0-29.9, 30+ g/d), smoking status (never, former, current 1-14/d, current 15-24/day, >=25/day), body mass index (<20, 20-24.9, 25-29.9, 30-34.9, 35+ kg/m2), physical activity (<3, 3-8.9, 9-17.9, 18-26.9, ≥27 METS), and dietary quality score (continuous score).

d. Model 4 included all covariates in model 3, and additionally adjusted for baseline cognitive function, mental health and psychological well-being including subjective cognitive decline (continuous score), depression diagnosis or antidepressant use (yes, no), depressive symptoms (continuous score), and optimism (continuous score).

© 2024 Chen Y et al. *JAMA Psychiatry.*

eTable 8. Complete-Case Analysis on the Associations Between Gratitude and All-Cause Mortality (Nurses' Health Study 2016-2019, N=32,706)

	Gratitude Score Tertiles		
	Bottom tertile (reference)	Middle tertile HR (95% CI)	Top tertile HR (95% CI)
Model 1 [a]	1.00	0.78 (0.71, 0.85)***	0.71 (0.65, 0.78)***
Model 2 [b]	1.00	0.80 (0.73, 0.88)***	0.74 (0.67, 0.81)***
Model 3 [c]	1.00	0.83 (0.75, 0.91)***	0.79 (0.72, 0.87)***
Model 4 [d]	1.00	0.93 (0.85, 1.03)	0.91 (0.82, 1.01)

Notes: HR, hazard ratio; CI, confidence interval. The analyses excluded participants with missing data on gratitude or any covariates. *p<0.05, **p<0.01, ***p<0.001.

a. Model 1 controlled for age (years), race/ethnicity (non-Hispanic white, others), marital status (married/in partnership, divorced/separated, widowed), geographic region (Northeast, Midwest, South, West), educational level (RN, bachelor, graduate), census-tract median household income (<$50,000, $50,000-$74,999, $75,000-$99,999, >=$100,000), census-tract college education rate (continuous), retired (yes, no), living arrangement (live alone, others), special residential setting (yes, no), social participation (none, 1-2 hours/week, 3-5 hours/week, 6-10 hours/week, 11+ hours/week), religious service attendance (never/almost never, <once/week, >=once/week), and religious coping (not at all, not very, somewhat, very).

b. Model 2 included all covariates in model 1, and additionally adjusted for baseline physical health status including history of heart disease (yes, no), stroke (yes, no), cancer (yes, no), hypertension (yes, no), hypercholesterolemia (yes, no), and diabetes (yes, no).

c. Model 3 included all covariates in model 2, and additionally adjusted for baseline health behaviors including alcohol intake (0, 0.1-9.9, 10.0-29.9, 30+ g/d), smoking status (never, former, current 1-14/d, current 15-24/day, >=25/day), body mass index (<20, 20-24.9, 25-29.9, 30-34.9, 35+ kg/m2), physical activity (<3, 3-8.9, 9-17.9, 18-26.9, ≥27 METS), and dietary quality score (continuous score).

d. Model 4 included all covariates in model 3, and additionally adjusted for baseline cognitive function, mental health and psychological well-being including subjective cognitive decline (continuous score), depression diagnosis or antidepressant use (yes, no), depressive symptoms (continuous score), and optimism (continuous score).

© 2024 Chen Y et al. *JAMA Psychiatry.*

eTable 9. Associations Between Covariates and All-Cause Mortality in the Fully Adjusted Model* (Nurses' Health Study 2016-2019, N=49,275)

	Hazard Ratio	95% Confidence Interval
Gratitude (lowest tertile as ref)		
Middle tertile	0.93	0.86, 1.01
Highest tertile	0.91	0.84, 0.99
Non-Hispanic white (other race/ethnicity as ref)	1.36	1.12, 1.64
Marital status (married or in partnership as ref)		
Divorced/separated	1.31	1.14, 1.50
Widowed	1.26	1.16, 1.38
Geographic region (northeast as ref)		
Midwest	1.10	1.02, 1.20
South	0.98	0.90, 1.06
West	0.94	0.86, 1.03
Educational level (RN as ref)		
Bachelor	1.01	0.82, 1.24
Graduate	1.09	0.90, 1.32
Census-tract household median income (<$50,000 as ref)		
$50,000-$74,999	0.98	0.90, 1.06
$75,000-$99,999	1.03	0.91, 1.17
>=$100,000	0.93	0.76, 1.13
Census-tract college education rate	0.95	0.68, 1.32
Retired (no as ref)	1.29	1.10, 1.50
Live alone (no as ref)	0.91	0.84, 0.98
Special residential setting (no as ref)	1.27	1.16, 1.39
Social participation (none as ref)		
1-2 hours/week	0.88	0.81, 0.96
3-5 hours/week	0.79	0.72, 0.86
6-10 hours/week	0.79	0.71, 0.89
11+ hours/week	0.74	0.64, 0.85
Religious service attendance (never/almost never as ref)		
Less than once/week	1.02	0.93, 1.13
At least once/week	0.85	0.78, 0.92
Religious coping (not at all as ref)		
Not very	1.01	0.89, 1.14
Somewhat	1.03	0.94, 1.14
Very	1.07	0.96, 1.18
Cigarette smoking (never as ref)		
Former smoker	1.25	1.17, 1.33
Current smoker, 1-14/day	1.83	1.52, 2.21
Current smoker, 15-24/day	1.42	1.06, 1.92
Current smoker, >=25/day	2.06	1.21, 3.53
Alcohol intake (0 grams/day as ref)		
0.1-9.9 grams /day	0.84	0.78, 0.91
10.0-29.9 grams /day	0.87	0.79, 0.96

© 2024 Chen Y et al. *JAMA Psychiatry.*

30+ grams /day	0.79	0.67, 0.92
Physical activity (<3 METS as ref)		
3-8.9 METS	0.84	0.77, 0.91
9-17.9 METS	0.75	0.69, 0.83
18-26.9 METS	0.68	0.61, 0.76
>=27 METS	0.59	0.53, 0.65
BMI categories (<20 kg/m^2 as ref)		
20-24.9 kg/m^2	0.60	0.55, 0.66
25-29.9 kg/m^2	0.50	0.46, 0.55
30-34.9 kg/m^2	0.52	0.46, 0.58
35+ kg/m^2	0.61	0.53, 0.72
Dietary quality score	0.99	0.99, 0.99
History of heart disease (no as ref)	1.28	1.19, 1.38
History of stroke (no as ref)	1.38	1.26, 1.52
History of cancer (no as ref)	1.45	1.36, 1.54
History of high cholesterol (no as ref)	0.91	0.85, 0.99
History of hypertension (no as ref)	1.20	1.11, 1.30
History of diabetes (no as ref)	1.35	1.26, 1.46
Optimism	1.03	1.02, 1.03
Depression diagnosis or antidepressant use (no as ref)	1.07	0.99, 1.16
Depressive symptoms	1.13	1.12, 1.14
Subjective cognitive decline	1.00	0.97, 1.02

*The analysis was stratified jointly by age in months and calendar time. Multiple imputation was performed to impute missing data on covariates. The effect estimates reported in this table correspond to Table 2 Model 4 (the fully-adjusted model).

© 2024 Chen Y et al. *JAMA Psychiatry.*
