# Supplementary material for: Gratitude and Mortality Among Older US Female Nurses
Source: JAMA Psychiatry. 2024 Jul 3;81(10):1030–8. doi: 10.1001/jamapsychiatry.2024.1687 (PMC11223047; doi:10.1001/jamapsychiatry.2024.1687)
Supplement: Supplement 2. — Data Sharing Statement [file jamapsychiatry-e241687-s002.pdf]

Data Sharing Statement

Chen. Gratitude and Mortality Among Older US Female Nurses. *JAMA Psychiatry*. Published July 03, 2024. doi:10.1001/jamapsychiatry.2024.1687

Data
Data available: No

Additional Information
Explanation for why data not available: Data of the Nurses' Health Study (NHS) are not publicly available. Further information including the procedures to obtain and access data from NHS is described at https://www.nurseshealthstudy.org/researchers (email: nhsaccess@channing.harvard.edu).
